# Crystallographic and optical study of LiNb_1 − *x*_Ta*_x_*O_3_

**DOI:** 10.1107/S2052520617004711

**Published:** 2017-06-01

**Authors:** S. Huband, D. S. Keeble, N. Zhang, A. M. Glazer, A. Bartasyte, P. A. Thomas

**Affiliations:** a Department of Physics, University of Warwick, Coventry, West Midlands CV4 7AL, England; b Diamond Light Source Ltd, Harwell Science and Innovation Campus, Chilton, Didcot, Oxfordshire OX11 ODE, England; c Electronic Materials Research Laboratory, Key Laboratory of the Ministry of Education and International Center for Dielectric Research, Xi’an Jiaotong University, Xi’an 710049, People’s Republic of China; d Physics Department, University of Oxford, Clarendon Laboratory, Parks Road, Oxford OX1 3PU, England; e FEMTO-ST Institute, University of Bourgogne Franche-Comté, CNRS, ENSMM, Time and Frequency Department, 26 rue de l’Epitaphe, 25030 Besançon, France

**Keywords:** lithium niobate tantalate, zero birefringence, metripol

## Abstract

Powders of lithium niobate-tantalate across the full compositional range have been made and crystals grown using a lithium vanadate flux growth technique. The Li-content of a lithium tantalate crystal has been determined using the zero-birefringence temperature and Curie measurements, confirming the Li content is between that of congruent and stoichiometric crystals. X-ray diffraction measurements show the Nb/Ta displacement and octahedral tilt both decrease as the Ta content is increased. This also results in a decrease in the lattice parameters from lithium niobate to lithium tantalate. Birefringence measurements on the crystals as a function of temperature have been used to determine the point that the crystals become zero-birefringent, and by comparison with the structural studies have confirmed that it is not related to a phase transition and the structures remain polar through the zero-birefringence points.

## Abstract 2

The structure of lithium niobate-tantalate has been studied using powder and single-crystal X-ray diffraction focusing on the composition and temperature induced zero-birefringence points.

## 1. Introduction   

Lithium niobate (LN) and lithium tantalate (LT) have both been used extensively for their optical properties in a range of devices: including optical modulators, Pockels cells, optical parametric oscillators, waveguides *etc*. LN and LT are isostructural and ferroelectric at room temperature, with space group *R*3*c*. Their physical properties, however, differ from one another. For example, at room temperature LN has a negative birefringence and congruent LT is positive. Congruent crystals form with the same composition as the initial materials, which does not occur at the stoichiometric (equal Li and Nb or Ta content) composition for LN or LT. In these materials the congruent compositions are Li-deficient with compositions around 48.45 mol% Li_2_O (O’Bryan *et al.*, 1985 ▸; Kushibiki & Ohashi, 2006 ▸).

A solid-solution LiNb_1 − *x*_Ta*_x_*O_3_ (LNT) can be formed across the whole compositional range and allows materials to be produced with properties intermediate between those of LN and LT, such as zero-birefringent crystals when the composition is LiNb_0.06_Ta_0.96_O_3_ (Peterson *et al.*, 1967 ▸; Shimura, 1977 ▸; Kondo *et al.*, 1979 ▸; Wood *et al.*, 2008 ▸). A better understanding of the physical properties as a function of composition could extend the use of current devices that are based solely on either LN or LT. For example, a patent for an LT-based device has been produced by Glazer *et al.* (2011 ▸) to accurately measure temperature in the vicinity of the zero-birefringence point. By changing the composition across the LNT solid solution the birefringence of the crystals can be varied allowing a larger operating range for the temperature sensor. The development of these devices relies on understanding the structural and physical properties across the compositional range.

A number of structural studies have been made on LN and LT, generally using congruent crystals that have been Czochralski grown (Abrahams, Reddy *et al.*, 1966 ▸; Abrahams, Hamilton *et al.*, 1966 ▸; Abrahams, Levinstein *et al.*, 1966 ▸; Abrahams & Bernstein, 1967 ▸; Abrahams *et al.*, 1967 ▸, 1973 ▸). The initial measurements of the physical properties varied between samples because they can be made with a range of Li content. LN can be made in the range of 46.4–50 mol% Li_2_O and LT in the range 46–50 mol% Li_2_O (Carruthers *et al.*, 1971 ▸; Barns & Carruthers, 1970 ▸). Both LN and LT undergo a high-temperature phase transition to a paraelectric structure with space group 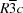, with the transition varying from 1310 to 1480 K and 780 to 965 K as the Li content increases to 50 mol% Li_2_O for LN and LT, respectively (Bordui *et al.*, 1995 ▸; Barns & Carruthers, 1970 ▸).

Structural models including the Li deficiency for LN were introduced by Lerner *et al.* (1968 ▸) and Abrahams & Marsh (1986 ▸) who suggested Li and Nb vacancies, respectively. The formula for the Li vacancy model is given by [Li_1 − 5*y*_ *M*_*y*_]*M*O_3_, and the Nb vacancy model is given by [Li_1 − 5*y*_ *M*_5*y*_]*M*_1 − 4*y*_O_3_. In the formulae the Li deficiency is given by *y* and *M* is the Nb content. In this work *M* also represents Ta and Nb/Ta mixtures. From these models the corresponding mol% Li_2_O required can then be calculated by mol% Li_2_O = Li/(Li + *M*). The correct Li deficiency model has not yet been satisfactorily determined and for this reason the Li content is commonly expressed using the mol% Li_2_O that would be required in the initial materials (Li_2_O and *M*_2_O_5_). This convention will be followed in this work when the Li content of materials is discussed. A set of parameters for rhombohedral perovskite structures such as LN and LT was introduced by Megaw & Darlington (1975 ▸) to parameterize the structure using the cation positions, octahedral distortion and octahedral tilt. In this system the O atom is held fixed at the first O plane (*z* = 1/12). The position of the Nb/Ta atom is then given by its displacement (*t*) along the *c*-axis from the origin, which is also the centre of the oxygen octahedra. The Li position is given by its displacement (*s*) from the O plane at *z* = 1/4 and the O position by the octahedral distortion (*d*) and tilt (*e*). In polar space groups, the octahedral distortion (*d*) is a displacement of the O atom in the *xy*-plane that allows the size of the upper and lower octahedral faces to differ. The upper face of the octahedra is smaller or larger when the octahedral distortion is positive or negative, respectively. The parameter *e* is related to the octahedral tilt ω by
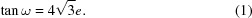



The change in these parameters with increasing temperature was calculated for LN by Megaw (1968*b* ▸) using the powder X-ray diffraction (XRD) measurements of Abrahams, Levinstein & Reddy (1966 ▸); the octahedral tilt decreases with increasing temperature, resulting in an increase in the *a* lattice parameter. The *c* lattice parameter initially increases until reaching a maximum and then decreases until the Curie point. The curve in the *c* lattice parameter is explained by the decrease in the Nb displacement with increasing temperature. In the paraelectric phase, both lattice parameters increase with temperature and the Nb displacement and octahedral distortion disappear; however, the octahedral tilt remains.

The birefringence of Czochralski-grown LNT as a function of composition has been studied by Wood *et al.* (2008 ▸) who showed that the birefringence increases linearly as the Ta concentration is increased and a sample with 94 mol% LT would have zero birefringence at room temperature. The properties of LNT are also dependent on the Li content of the crystals; the change in the Curie point and zero-birefringence point have previously been used as a way to quantify the Li content of LT crystals (Barns & Carruthers, 1970 ▸; Bäumer *et al.*, 2003 ▸). The birefringence of LNT crystals is dependent on both the LN/LT content and the Li content, with an increase in Li or LN content resulting in a decrease in the birefringence. A detailed structural investigation has not been made focusing on the zero-birefringence points of LN, LT or LNT crystals.

LNT thin films on LT substrates were produced using a LiVO_3_ (LV) flux-growth technique by Kondo *et al.* (1979 ▸), who also produced a phase diagram showing the super-saturation temperature as a function of both the LNT composition and LV content. Recently crystals of LNT have been grown using this flux-growth technique and compared to those made using Czochralski, top-seeded solution growth (TSSG) and floating zone methods (Bartasyte *et al.*, 2012 ▸). Crystals grown using these four methods resulted in crystals which were Ta-rich compared with the initial materials and considerable Li loss was observed for crystals grown using the floating zone technique.

Here the structures across the LNT compositional range are reported, whilst also considering the Li content of the samples used. XRD measurements were made on a series of powder samples to determine the change in lattice parameters as a function of LT content and temperature. Using these powders as the starting materials, crystals were grown using a LV flux. XRD was used to calculate the structures and the birefringence was then investigated as a function of temperature with a focus on the zero-birefringence points.

## 2. Experimental details   

Powder LNT samples with a mass of 15 g were prepared by a solid-state reaction between Li_2_CO_3_ (99.99%), Nb_2_O_5_ (99.99%) and Ta_2_O_5_ (99.85%). The starting materials were weighed according to the desired ratios and then ball-milled in iso­propanol, placed in a platinum crucible with a lid and sintered for 140 h at 1433 K, following the method used by Bartasyte *et al.* (2012 ▸). The main set of samples were made with compositions (LiNb_1 − *x*_Ta_*x*_O_3_) of *x* = 0, 0.1, 0.2, 0.3, 0.4, 0.5, 0.6, 0.7, 0.8, 0.9 and 1, with a further focused region every 0.01 between 0.9 and 1. Variable-temperature measurements were made on compositions with *x* = 0.94, 0.96, 0.98, 0.99 and 1, along with high-temperature measurements only on *x* = 0.92.

Low-temperature X-ray diffraction measurements on the powders were performed using a Bruker D5005 diffractometer equipped with a Cu *K*α source and an Oxford Cryosystems Phenix cryostat. Room- and high-temperature XRD measurements were made using a PANalytical MPD diffractometer using Cu *K*α_1_ radiation and an Anton Paar HTK1200 sample furnace. A reflection spinner stage was used for room-temperature measurements with a sample diameter of 1.6 cm. The measurements made with the Anton Paar HTK1200 were also made in air using a sample holder with a diameter of 1.6 cm. Measurements between 20 and 135° in 2θ were made every 20 K between 40 and 300 K and every 40 K between 300 and 1100 K. All sintered powders were ground using a pestle and mortar prior to the XRD measurements. Long room-temperature measurements were made on all of the main samples along with *x* = 0.91, 0.93, 0.95 and 0.99 from the focused region.

Crystals of LNT were grown using a LV flux and a three-zone vertical tube furnace. A molar ratio of 10 moles of LV to 1 mole of LiNb_1 − *x*_Ta_*x*_O_3_ was used for the crystal growth of all compositions. The mixed powders were placed in a platinum crucible and covered with a platinum lid. They were heated at a rate of 180 K h^−1^ to 1473 K, followed by 20 K h^−1^ to 1573 K and held for 24 h to homogenize the melted material. This was then cooled at a rate of 1 K h^−1^ until the super-saturation point of the LiNb_1 − *x*_Ta_*x*_O_3_ in LV given by the LNT–LV phase diagram (Kondo *et al.*, 1979 ▸). Once the temperature was sufficiently below the super-saturation point, the cooling rate was increased to 60 K h^−1^.

The as-grown orientations of the LNT crystals were measured using a PANalytical X’Pert Pro MRD system, equipped with a 4-bounce hybrid monochromator to provide an intense source of Cu *K*α_1_ radiation and a PIXcel detector. The chemical composition of the samples was studied using energy-dispersive X-ray analysis (EDX) on a Zeiss Supra 55VP FEG scanning electron microscope with EDAX GENESIS software. Single-crystal X-ray diffraction measurements of the grown crystals were performed using an Oxford diffraction Gemini diffractometer equipped with a Mo *K*α source.

The experimental setup for second harmonic generation (SHG) measurements is based on that of Kurtz & Perry (1968 ▸) and is described fully in Jones & Thomas (2002 ▸). A Nd-YAG laser with a wavelength of 1064 nm and a modulated frequency of 1 Hz was used. The beam was initially split with a glass slide, with one beam sent to a photodiode and the other directed at a sample held inside a furnace with two holes to allow light to pass through. The SHG signal with a wavelength of 532 nm was measured using a photomultiplier tube (PMT) positioned behind a green filter to ensure only the second-harmonic signal was detected. An oscilloscope was used to measure the pulses from the photodiode and the PMT and a computer running LabVIEW was used to record the data from the oscilloscope and calculate an average signal from 10 pulses.

Birefringence imaging microscopy (BIM; Glazer *et al.*, 1996 ▸) measurements were made every 1 K, while heating and cooling the crystal at a rate of 60 K h^−1^ between 300 and 873 K using a Linkam THMS600 hot-stage. In the BIM setup, the measured intensity of the rotating polariser and circular analyser is given by
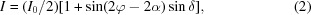
where *I*_0_ is equivalent to the transmitted light intensity, φ is the angle measured anticlockwise between the horizontal axis of the microscope (given by the top and bottom of the CCD array of the detector) and the slow axis of the indicatrix, α is the angle of the polariser from a predetermined zero, and δ is the phase shift of the light given by
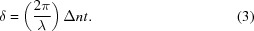



Δ*n* is the observed birefringence, *t* is the sample thickness and λ is the light wavelength. The BIM measurements were analysed using the Metripol software, which produces false-colour images of |sinδ|, φ and the transmitted intensity. The measured |sin δ| oscillates between 0 and 1, with a peak when 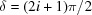 and a trough when 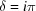, where *i* is an integer. To determine which trough corresponds to the material being zero-birefringent (*i* = 0) usually requires separate measurements of the refractive indices, however, this is not required when the variation in the birefringence as a function of wavelength is small. In this case, the zero-birefringence (*i* = 0) troughs will be at the same temperature at each wavelength. Troughs that occur when δ is π, 2π *etc.* will not be at the same temperature for different wavelengths, allowing the trough corresponding to zero-birefringence to be determined. It has been shown that the birefringence measured between 589 and 1060 nm is relatively constant in LNT (Shimura, 1977 ▸), allowing the zero-birefringence point to be determined with the use of 550, 570, 590 and 600 nm wavelength filters.

## 3. Results and discussion   

### 3.1. Powder diffraction   

**Figure 1:**
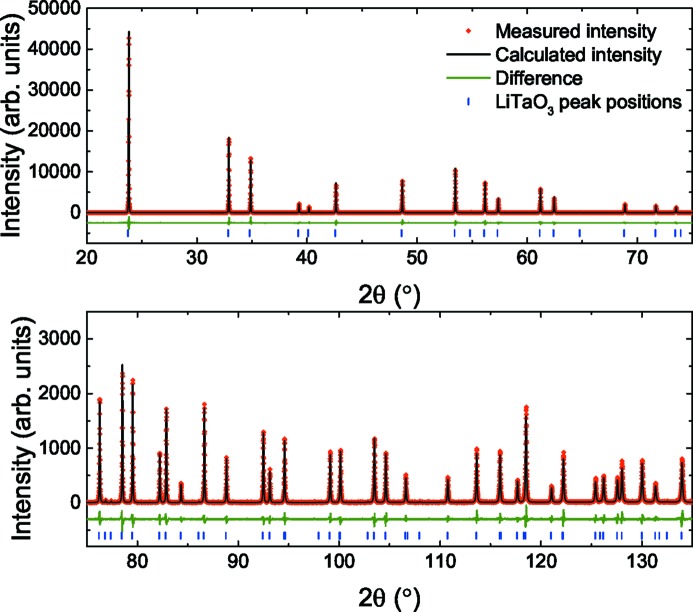
Rietveld refinement of the powder XRD measurements on LT.

Rietveld refinements of the room-temperature XRD measurements were made using *TOPAS Academic* (Coelho, 2007 ▸). The powders were phase-pure and the refinement of the LT sample is shown in Fig. 1 ▸. The measured lattice parameters and Megaw parameters are given in Table 1 ▸ along with those calculated by Megaw & Darlington (1975 ▸) using the powder X-ray diffraction and single-crystal neutron diffraction measurements of Abrahams & Bernstein (1967 ▸) and Abrahams *et al.* (1967 ▸). The crystal used by Abrahams *et al.* was Czochralski-grown using a congruent composition, whereas the powder sample in this study was stoichiometric. The lattice parameters of the stoichiometric powder are lower than those of the congruent crystal, consistent with the results of Barns & Carruthers (1970 ▸). For all compositions, there are no visible superlattice reflections from the ordering of Nb and Ta atoms.

**Figure 2:**
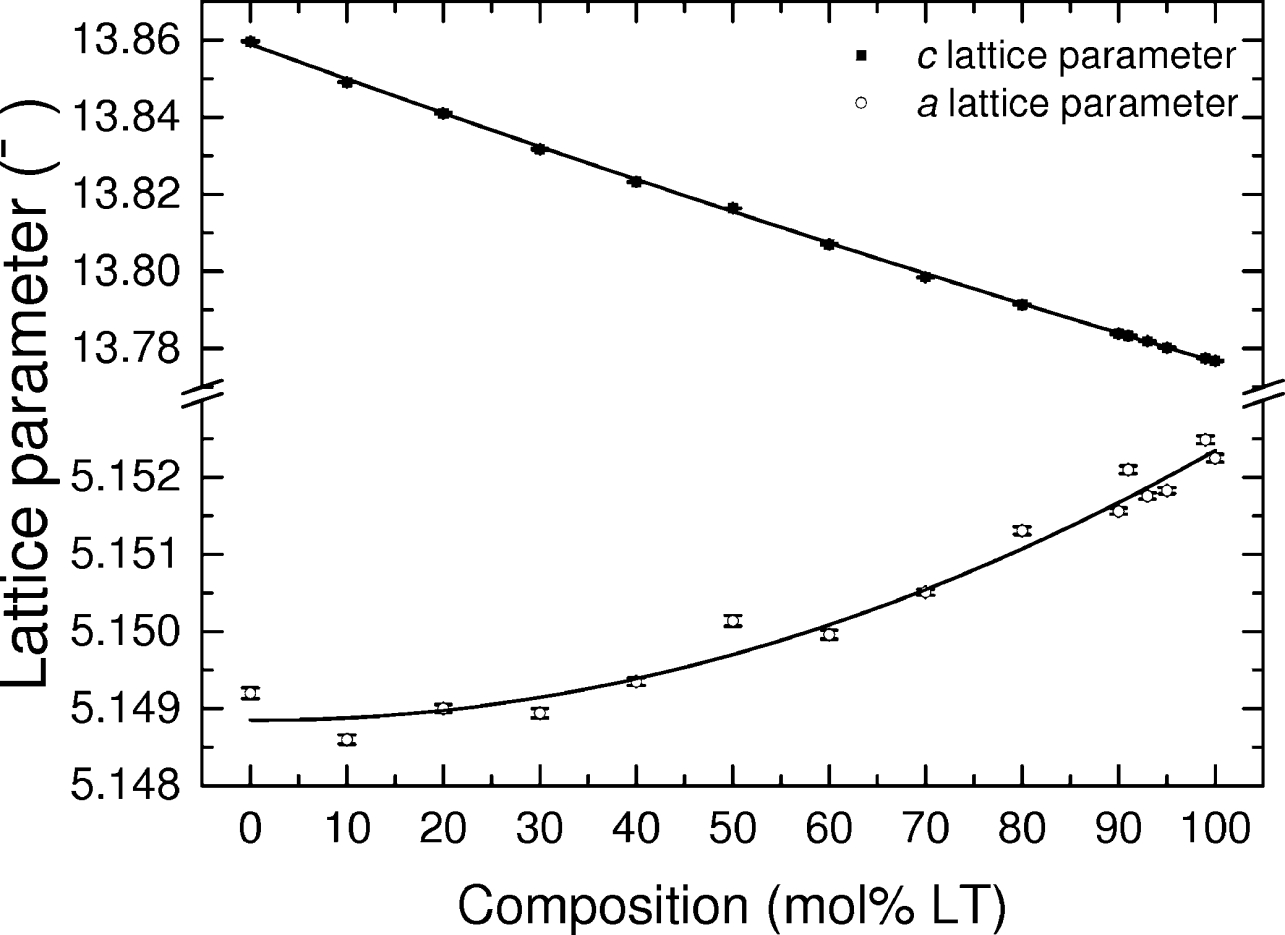
The *a* and *c* lattice parameters from powder XRD measurements as a function of LT content. Quadratic fits are given by the lines.

It was found that LNT samples with less than 70 mol% LT content formed with preferred orientation in the (012) plane. The effect of the preferred orientation on the measured diffraction profiles made the refinement of the atomic positions unreliable. The refined lattice parameters are unaffected by this and are shown in Fig. 2 ▸. The *a* and *c* lattice parameters both show a deviation from Vegard’s law. They have been fitted with a quadratic function containing a bowing parameter (*b*) to describe the deviation
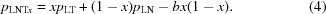
where *x* is the Ta content in LiNb_1 − *x*_Ta_*x*_O_3_ and *P*_LNT_, *P*_LT_ and *P*_LN_ are the lattice parameters of LNT, LT and LN, respectively. The *a* lattice parameter has a bowing parameter of −0.0036 (8), which agrees with the previously determined value of −0.003 (3) (Bartasyte *et al.*, 2012 ▸). The *c* lattice parameter has a bowing parameter of −0.009 (2), which is less than the previously determined value of −0.016 (3). This difference is likely to be a result of the increased number of samples measured with low-LT content in this study. The change in lattice parameter as a function of composition has also been studied by Bak *et al.* (2014 ▸) who showed a similar decrease in the *c* lattice parameter with increasing Ta content. However, they determined the *a* lattice parameter changes to be negligibly small. The uncertainties in the Megaw *t*, *s*, *d* and tilt parameters are too high to determine a trend in this compositional range.

**Figure 3:**
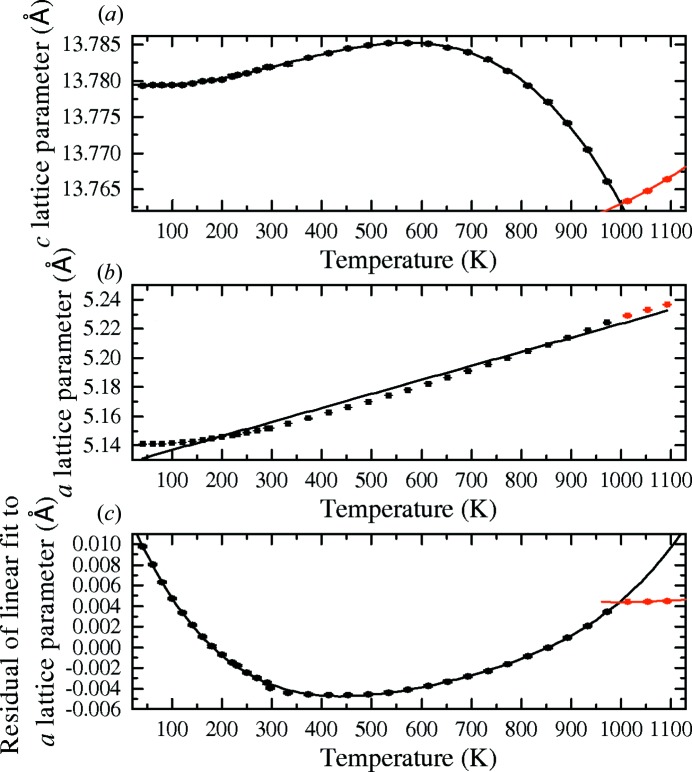
The (*a*) *c* lattice parameter and (*b*) *a* lattice parameters as a function of temperature for an LNT94 powder sample. In (*b*), the black line gives a linear fit to the data, and the residual of this fit is plotted in (*c*). The data above the Curie point are red and have been fitted with a second-order polynomial in (*a*) and (*c*), below the Curie point the points are black and are fitted by a fourth-order polynomial in (*a*) and (*c*).

The lattice parameters of high Ta-content powders have been investigated using XRD measurements between 40 and 1100 K. The *c* lattice parameter for LiNb_0.06_Ta_0.94_O_3_ is plotted in Fig. 3 ▸(*a*) along with the *a* lattice parameter in Fig. 3 ▸(*b*). The variation with temperature of the lattice parameters in LiNb_0.06_Ta_0.94_O_3_ is consistent with the powder XRD measurements of Sugii *et al.* (1976 ▸) who studied a range of compositions between 300 and 1273 K using crushed Czochralski grown crystals. The lower temperatures studied here show that the lattice parameters decrease less as the temperature is reduced below 250 K and start to level off.

The *c* lattice parameter increases from 40 to 600 K and then decreases to lower than the initial value at 40 K. It decreases until the discontinuity at the Curie point and then begins increasing in the paraelectric phase. The peak in the *c* lattice parameter is also evident in all compositions measured by Sugii *et al.* (1976 ▸) and is consistent with the suggestion of Megaw (1968*b* ▸) that the peak and resultant decrease in the *c* lattice parameter is a result of the decrease in the Nb/Ta displacement. The measured lattice parameters have been fitted with second- and fourth-order polynomials above and below the Curie point, respectively.

The *a* lattice parameter as a function of temperature is plotted in Fig. 3 ▸(*b*) and has a non-linear increase with temperature. There is also a discontinuity at the Curie point which can be seen clearly by plotting the residual of a linear fit to the data, as shown in Fig. 3 ▸(*c*). As with the *c* lattice parameter, second- and fourth-order polynomials have been used to fit the residual above and below the Curie point, respectively. The crossing of the polynomial fits provides a measurement of the Curie point of the powder sample. For the LiNb_0.06_Ta_0.94_O_3_ sample this corresponds to 999 (3) K.

**Figure 4:**
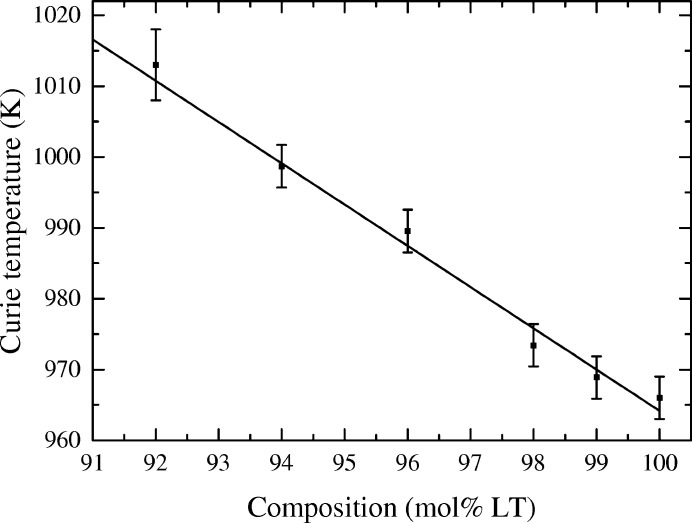
The Curie point of Ta-rich LNT powders as a function of LT content.

The Curie point as a function of composition for samples with greater than 90 mol% LT is plotted in Fig. 4 ▸. In this small compositional range the Curie point increases linearly as the Ta concentration is decreased, in agreement with the trend determined by Peterson *et al.* (1970 ▸). The LT sample has a Curie point at 966 (3) K, which agrees with the value of 963 K determined on a stoichiometric crystal by Barns & Carruthers (1970 ▸).

### 3.2. LNT crystal measurements   

The crystals grown using the flux-growth technique were predominantly plate-like with a few cuboidal crystals. Many of the crystals were visibly twinned and contained flux inclusions. Following each growth, the best-quality crystal was selected and prepared for further measurements. The crystals were polished such that the largest as-grown face became one surface of a crystal plate between 200 and 300 µm thick. For the congruent crystal measurements, a Z-cut plate of LT purchased from MTI Corporation (a supplier of crystal substrates) was used.

**Figure 5:**
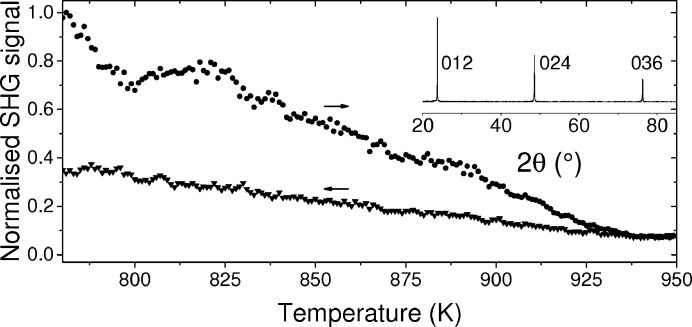
The normalized SHG signal during heating and cooling of a flux-grown LT crystal and inset is a 2θ XRD scan, showing the 012, 024 and 036 reflections.

The normalized SHG signal for the LNT100 crystal, grown by the LV flux method, is plotted during heating and cooling in Fig. 5 ▸. The measured signal is lower on cooling than on heating and both drop to a background level at the Curie point. This was judged visually along with the uncertainties giving a Curie point of 940 (4) K. The Curie point of the congruent LT crystal is 873 (4) K and closely agrees with the value of 876.0 K precisely determined by Kushibiki & Ohashi (2006 ▸). The Li content of the LT sample is 49.65 (10) mol% Li_2_O according to the relationship between Curie temperature and composition given by Bordui *et al.* (1995 ▸). This gives the formula Li_0.98675_(Nb_1 − *x*_Ta_*x*_)_1.00265_O_3._ This composition is between the congruent and stoichiometric values of 48.6 and 50 mol% Li_2_O, respectively. There is likely to be some variation in the Li content of the crystals at different LN/LT compositions, but it is expected to remain between the congruent and stoichiometric values. It was shown for LT by Barns & Carruthers (1970 ▸) that a decrease in Li content would cause an increase in the *a* and *c* lattice parameters.

The planes corresponding to the large surfaces of all of the LNT flux grown plates are the (012) planes, as shown by the out-of-plane XRD measurements inset in Fig. 5 ▸. These scans include three peaks corresponding to the 012, 024 and 036 reflections. This is consistent with the preferred orientation of the powder LNT samples, also, these planes are the twin planes and dominant cleavage planes in LN and LT.

The composition of the grown crystals has been determined using EDX and is in agreement with the results of Bartasyte *et al.* (2012 ▸) that the crystals are Ta-rich compared with the initial materials. The determined compositions are listed in Table 2 ▸ and the uncertainties were estimated from the variation in the values measured across the samples. There was no evidence from the EDX of V contamination within the crystals, however, the crystals had a yellow/green coloration. LN and LT crystals are clear, which suggests there could be some V contamination, which is below the detection limits of EDX.

The collected diffraction data were indexed and integrated using the *CrysAlisPro* software and the absorption corrected with the analytical method of Clark & Reid (1995 ▸). The corrected intensities were analysed using *SHELX* and the refined structures from the powder diffraction measurements were used as a starting point. Initial refinements were made allowing the Nb and Ta occupancies to vary, but these were not in agreement with the values determined by EDX and varied inconsistently as a function of composition. Following this, the refinements were made with fixed Nb and Ta occupancies similar to the Rietveld refinements. Attempts were also made to refine the data using the Li-deficiency models but these were also unsuccessful, so the refinements were made with the occupancies fixed at their stoichiometric values.

**Figure 6:**
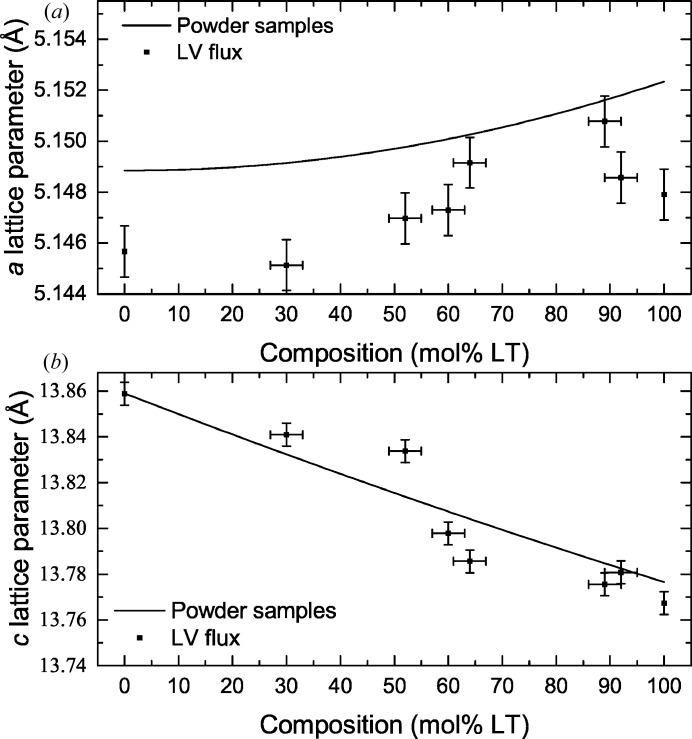
The (*a*) *a* lattice parameters and (*b*) *c* lattice parameters of single-crystal LV flux-grown LNT crystals as a function of composition. The lines are the quadratic fits to the powder samples in Fig. 2 ▸.

Structural refinements using *SHELX* gave *R*_obs_ values between 0.014 and 0.025, with anisotropic displacement parameters (Sheldrick, 2008 ▸). The *a* and *c* lattice parameters are plotted as a function of the measured composition of the crystals in Fig. 6 ▸, along with the results of the powder diffraction measurements. For the *c* lattice parameter, the measurements on powders and single crystals agree with each other. The *a* lattice parameter values from single crystals are less than those from powder measurements. This could be the result of a small amount of V incorporation within the crystals, which is below the detection limits of the EDX measurements.

The refined Megaw parameters for the LNT single crystals are included in Table 2 ▸, along with the previous values determined for LN and LT (Abrahams, Reddy *et al.*, 1966 ▸; Abrahams, Hamilton *et al.*, 1966 ▸; Abrahams & Bernstein, 1967 ▸; Abrahams *et al.*, 1967 ▸). The values determined using LV flux-grown crystals agree with those determined previously on LN and LT crystals.

**Figure 7:**
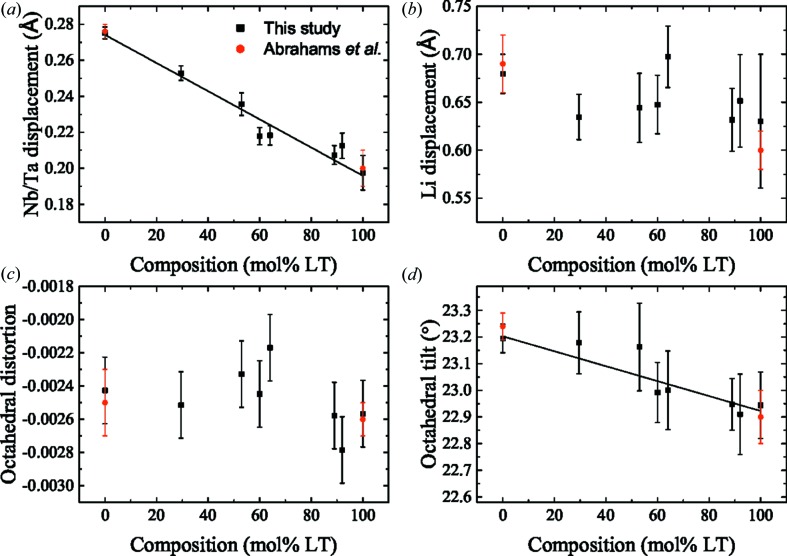
The (*a*) Nb/Ta displacement, (*b*) Li displacement, (*c*) octahedral distortion and (*d*) octahedral tilt of LV flux-grown LNT single crystals as a function of LT content, given by the black squares. The results previously calculated from X-ray and neutron diffraction measurements are given by the red circles (Abrahams & Bernstein, 1967 ▸; Abrahams *et al.*, 1967 ▸; Abrahams, Reddy *et al.*, 1966 ▸; Abrahams, Hamilton *et al.*, 1966 ▸).

The refined Megaw parameters of the LNT crystals are plotted in Fig. 7 ▸. The Nb/Ta displacement increases linearly as the Ta concentration is decreased. A trend in the Li displacement cannot be determined because of the uncertainties on the refined values. However, for all LNT compositions, the Li displacement is between 0.62 and 0.7 Å, which is consistent with values determined for LN and LT using neutron diffraction (Abrahams, Hamilton *et al.*, 1966 ▸; Abrahams *et al.*, 1967 ▸). The trend in the octahedral distortion also cannot be determined because of the measurement uncertainties, but it can be seen that the values are small and negative as expected from the previous measurements. The higher uncertainties in the Li and O position compared with those of Nb and Ta are expected because of their lower atomic scattering factors. However, despite the larger uncertainties for the position of the O atom, a trend showing an increase in the octahedral tilt with increasing LN content can be determined. The lack of scatter around the linear fits of the Nb/Ta displacement and octahedral tilt suggests a similar Li deficiency across the compositional range. The Li and O positions can be determined precisely using neutron diffraction, which has been done for LT as a function of temperature showing the Li displacement decreases with increasing temperature (Huband *et al.*, 2017 ▸).

It was shown by Megaw that the Nb displacement and octahedral tilt decrease with increasing temperature (Megaw, 1968*a* ▸,*b* ▸). This is similar to the change in these parameters as a function of composition; the decrease in the Ta content results in an increase in both the Nb/Ta displacement and the octahedral tilt. The increase in the Nb/Ta displacement also results in an increase in the *c* lattice parameter, whereas the increase in the octahedral tilt angle results in a decrease in the *a* lattice parameter.

**Figure 8:**
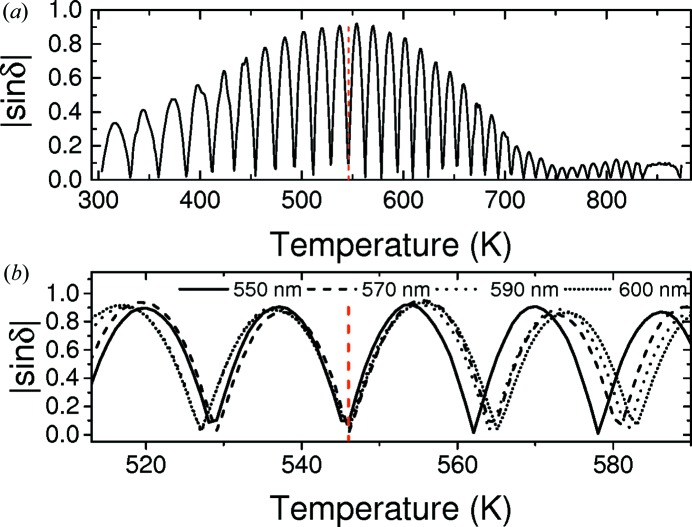
The measured |sin δ| of the flux-grown LiNb_0.34_Ta_0.64_O_3_ crystal while heating using (*a*) 550 nm and (*b*) 550, 570, 590 and 600 nm wavelength filters. The zero-birefringence temperature is given by the dashed vertical red line.

Heating and cooling runs were made using the Metripol system at 550, 570, 590 and 600 nm. The measured |sin δ| images of the LNT crystals were analysed by averaging the values measured in a 12 × 12 pixel square, sufficiently away from any scratches on the sample. The sample movement was accounted for to ensure the same sample area was analysed at each temperature. The measured |sin δ| at 550 nm as a function of temperature for the LiNb_0.34_Ta_0.64_O_3_ crystal is plotted in Fig. 8 ▸(*a*) during heating. Theoretically, the |sin δ| signal should oscillate between 0 and 1; however, this only occurs at the zero-birefringence point and the amplitude of the oscillations decreases above and below this. A similar effect has been measured in LT, LNT and other crystals that contain very small domains, which tend to scatter the light (Glazer *et al.*, 2010 ▸). The maximum operating temperature of the furnace is 873 K, limiting the range of composition that can be analysed with this technique.

**Figure 9:**
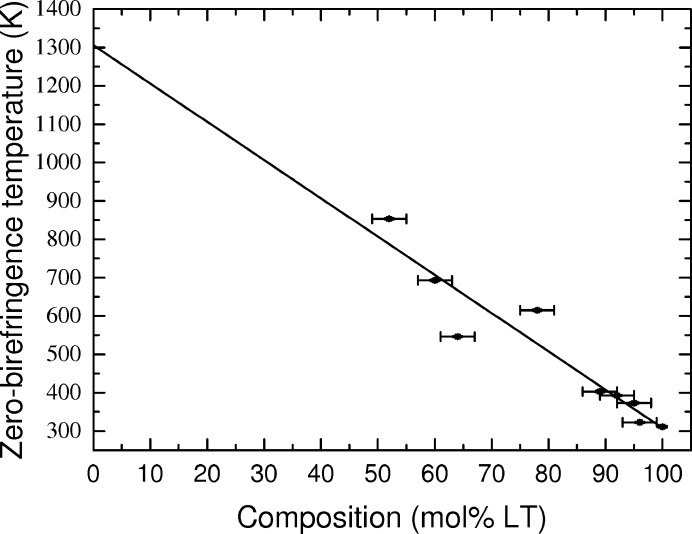
Zero-birefringence temperature of LNT crystals as a function of LT content.

The measured |sin δ| for each wavelength is shown in Fig. 8 ▸(*b*) for LiNb_0.34_Ta_0.64_O_3_. The trough corresponding to zero-birefringence is marked by the dashed vertical red line. Above and below this the troughs in each wavelength diverge allowing the zero-birefringence temperature to be determined. For the LV flux-grown crystals the measured zero-birefringence temperature increases linearly as the Ta concentration is decreased, shown in Fig. 9 ▸. This trend is consistent with the measurements of Wood *et al.* (2008 ▸) on congruent LNT crystals. The zero-birefringence value for the LT crystal is higher than the values measured on congruent samples, confirming that these crystals have a higher Li-content (Bäumer *et al.*, 2003 ▸). Using the comparison between Li content and zero-birefringence temperature produced by Bäumer *et al.* (2003 ▸) gives a Li content of 49.6 (1) mol% Li_2_O, which is in close agreement with the composition determined using the Curie point.

## 4. Conclusions   

The Li content of a LV flux-grown LT crystal was calculated to be 49.65 (10) and 49.6 (1) mol% Li_2_O using measurements of the Curie point and zero-birefringence temperature, respectively. The zero-birefringent temperature determined using flux-grown crystals increases as the LT content decreases, with a zero-birefringence temperature between 300 and 400 K for crystals with greater than 90 mol% LT. XRD and SHG measurements do not show any changes through these zero-birefringence points, confirming that the material remains polar at this temperature. At higher temperatures, the lattice parameters determined using high-resolution powder XRD measurements can be used to determine the transition temperature to the paraelectric phase.

Along with the zero-birefringence temperature, the *c* lattice parameter, Nb/Ta displacement and octahedral tilt angle also increase as the LT content is decreased. This compositional variation is similar to the thermal behaviour in LN as the temperature is increased; the *c* lattice parameter, Nb displacement and octahedral tilt decrease, whereas the birefringence increases (Megaw, 1968*b* ▸). This suggests the birefringence correlates with the structural changes; increases in the *c* lattice parameter, Nb/Ta displacement and octahedral tilt cause a decrease in the birefringence and result in a higher zero-birefringence temperature as the LT content is reduced.

These results also show that zero-birefringence in materials in which the birefringence has a small dependence on wavelength can be determined by repeated measurements using different wavelengths. This reduces the need to use compensation methods or more complicated methods to determine the order of the birefringence.

## Supplementary Material

Crystal structure: contains datablock(s) LNT0, LNT30, LNT53, LNT60, LNT64, LNT89, LNT92, LNT100. DOI: 10.1107/S2052520617004711/bp5094sup1.cif

Structure factors: contains datablock(s) LNT0. DOI: 10.1107/S2052520617004711/bp5094LNT0sup2.hkl

Structure factors: contains datablock(s) LNT30. DOI: 10.1107/S2052520617004711/bp5094LNT30sup3.hkl

Structure factors: contains datablock(s) LNT53. DOI: 10.1107/S2052520617004711/bp5094LNT53sup4.hkl

Structure factors: contains datablock(s) LNT60. DOI: 10.1107/S2052520617004711/bp5094LNT60sup5.hkl

Structure factors: contains datablock(s) LNT64. DOI: 10.1107/S2052520617004711/bp5094LNT64sup6.hkl

Structure factors: contains datablock(s) LNT89. DOI: 10.1107/S2052520617004711/bp5094LNT89sup7.hkl

Structure factors: contains datablock(s) LNT92. DOI: 10.1107/S2052520617004711/bp5094LNT92sup8.hkl

Structure factors: contains datablock(s) LNT100. DOI: 10.1107/S2052520617004711/bp5094LNT100sup9.hkl

CCDC references: 1540402, 1540403, 1540404, 1540405, 1540406, 1540407, 1540408, 1540409

## Figures and Tables

**Table 1:** Rietveld refinement results for LNT powders between 70 and 100 mol% LT

Composition (mol% LT)	*a* = *b* (Å)	*c* (Å)	*t* (Å)	*s* (Å)	*d*	Tilt (°)	*R*_wp_	*R*_p_	GOF
LT crystal†	5.15428 (1)	13.78351 (2)	0.20 (1)	0.60 (2)	−0.0026 (1)	22.9 (1)	–	–	–
LiTaO_3_	5.1523 (1)	13.7768 (1)	0.198 (4)	0.58 (1)	−0.0029 (3)	22.93 (8)	0.1468	0.0937	1.489
99	5.1525 (1)	13.7774 (2)	0.213 (4)	0.63 (1)	−0.0041 (2)	23.24 (7)	0.1093	0.0750	1.724
95	5.1518 (1)	13.7802 (2)	0.204 (3)	0.62 (1)	−0.0027 (2)	23.27 (6)	0.0949	0.0631	1.640
93	5.1518 (1)	13.7819 (2)	0.200 (3)	0.63 (1)	−0.0027 (2)	23.25 (6)	0.0954	0.0636	1.675
91	5.1521 (1)	13.7833 (2)	0.212 (3)	0.64 (1)	−0.0026 (2)	23.29 (6)	0.0894	0.0602	1.461
90	5.1516 (1)	13.7838 (2)	0.209 (3)	0.66 (1)	−0.0029 (3)	23.16 (6)	0.1068	0.0693	1.472
80	5.1513 (1)	13.7914 (2)	0.216 (3)	0.63 (1)	−0.0030 (3)	23.35 (6)	0.1109	0.0723	1.369
70	5.1505 (1)	13.7985 (2)	0.240 (2)	0.61 (1)	−0.0041 (2)	23.45 (5)	0.0835	0.0595	1.733

† Calculated by Megaw & Darlington (1975 ▸) using X-ray diffraction and single-crystal neutron diffraction measurements (Abrahams & Bernstein, 1967 ▸; Abrahams *et al.*, 1967 ▸).

**Table 2:** Refined structural parameters of flux-grown LNT crystals using XRD measurements

Composition (mol% LT)	*a* = *b* (Å)	*c* (Å)	*t* (Å)	*s* (Å)	*d*	Tilt (°)	*R*_obs_
LT crystal†	5.15428 (1)	13.78351 (2)	0.20 (1)	0.60 (2)	−0.0026 (1)	22.9 (1)	–
LT	5.148 (1)	13.767 (5)	0.208 (8)	0.62 (6)	−0.0027 (2)	22.9 (1)	0.0165
92 (3)	5.149 (1)	13.781 (5)	0.207 (7)	0.66 (4)	−0.0028 (2)	22.9 (1)	0.0190
89 (3)	5.151 (1)	13.776 (5)	0.203 (7)	0.62 (7)	−0.0026 (2)	22.9 (1)	0.0169
64 (3)	5.149 (1)	13.786 (5)	0.222 (6)	0.68 (4)	−0.0022 (2)	23.0 (1)	0.0155
60 (3)	5.147 (1)	13.798 (5)	0.216 (6)	0.65 (3)	−0.0024 (2)	23.0 (1)	0.0144
53 (3)	5.147 (1)	13.834 (5)	0.237 (8)	0.66 (6)	−0.0024 (2)	23.1 (1)	0.0235
30 (3)	5.145 (1)	13.841 (5)	0.255 (6)	0.64 (4)	−0.0025 (2)	23.2 (1)	0.0246
LN	5.146 (1)	13.859 (5)	0.276 (4)	0.69 (3)	−0.0025 (2)	23.24 (5)	0.0210
LN crystal†	5.14829 (2)	13.8631 (4)	0.28 (1)	0.70 (2)	−0.0025 (2)	23.3 (1)	–

† Calculated using X-ray and neutron diffraction measurements (Abrahams & Bernstein, 1967 ▸; Abrahams *et al.*, 1967 ▸; Abrahams, Reddy *et al.*, 1966 ▸; Abrahams, Hamilton *et al.*, 1966 ▸).
